# The INGENIA project and “Healthy Future”: a multidisciplinary innovative impulse within the SDGs

**DOI:** 10.1590/0034-7167-2024-0039

**Published:** 2025-12-08

**Authors:** Francisco Javier Castro-Molina, Gilberto Tadeu Reis da Silva, Sagrario Gómez-Cantarino, Jesús Manuel García-Acosta

**Affiliations:** I Universidad de La Laguna, Escuela Enfermería Nuestra Señora de Candelaria. Canary Islands, Spain; II Universidade Federal da Bahia, Salvador, Bahía, Brazil; III Universidad de Castilla La Mancha. Toledo, Spain

**Keywords:** Learning, Healthy Lifestyle, Students, Teachers, Primary and Secondary Education., Aprendizagem, Estilo de vida saudável, Alunos, Professores, Ensino básico e secundário.

## Abstract

**Objective:**

to evaluate the educational actions proposed in the field of healthy lifestyles developed by the Canary Islands Health Service and by the teaching staff of the educational centres, with the help of students of the Nursing degree using Service-Learning.

**Method:**

intervention project with a pretest-posttest that was developed from the II Edition of ‘INGENIA. Agents of change for the SDGs’, between February and July 2021. We proceeded to measure the impact of the intervention and the satisfaction of those involved.

**Results:**

The programme was a success, increasing both the knowledge of healthy habits of primary school pupils and the satisfaction of the agents implementing the programme.

**Final conclusions:**

the considerable increase in the satisfaction of both pupils and teachers who use this teaching methodology stands out, as well as the benefits it generates for the society in which they are involved.

## RESUMO

**Objetivo:**

avaliar as acções educativas propostas no âmbito dos estilos de vida saudáveis desenvolvidas pelo Serviço de Saúde das Canárias e pelo pessoal docente dos centros educativos, com a ajuda de estudantes do curso de Enfermagem, utilizando a Aprendizagem em Serviço.

**Método:**

projeto de intervenção com um pré-teste-pós-teste que foi desenvolvido a partir da II Edição do “INGENIA. Agentes de mudança para os ODS’, entre fevereiro e julho de 2021. Procedeu-se à medição do impacto da intervenção e da satisfação dos envolvidos.

**Resultados:**

O programa foi um sucesso, aumentando quer os conhecimentos sobre hábitos saudáveis dos alunos do ensino básico, quer a satisfação dos agentes que implementaram o programa.

**Conclusões finais:**

destaca-se o aumento considerável da satisfação tanto dos alunos como dos professores que utilizam esta metodologia de ensino, bem como os benefícios que gera para a sociedade em que estão inseridos.

## INTRODUCTION

The World Health Organisation (WHO) defines health as “a state of complete physical, mental and social well-being, and not merely the absence of disease or infirmity”. Therefore, we currently speak about healthy lifestyles, leaving behind the outdated concept of healthy life and paying attention to fundamental aspects such as physical exercise, nutrition, connections with the closest environment and the relationships we establish in our respective social niches. However, how we define “healthy lifestyle”? In three words: “living in harmony”. Namely, enjoying the best quality of life we can attain. Elements such as balanced nutritional intake, habitual physical exercise, having a healthy Body Mass Index (BMI), avoiding toxic substances, preserving ‘mental balance’ and sleeping the necessary hours become the required ingredients to achieve it^(1-3)^.

Both boys’ and girls’ health is fundamental so that they can lead a full life that allows them to achieve self-fulfilment in the future. Children’s health is grounded on three basic elements: early detecting developmental problems; preventing any contagious diseases that might emerge; and promoting healthy habits by means of interventions that foster Health Education (HE). Therefore, childhood is a crucial moment for learning where boys and girls can easily adopt healthy habits that will certainly remain in adulthood. In addition, it is to be noted that healthy habits prevent diseases that would otherwise appear as years go by, thus helping achieve balance from spheres such as the physical, psychological, social or spiritual ones. The Tenerife island is host to countless initiatives from the Primary Health Care network seeking to meet the population’s needs. In the case of children, they sometimes require close educational interventions within academic spaces in teaching centres, where they should be instructed on the basic habits that ease implementing a healthy lifestyle,^(2-6)^.

Developed in the University of La Laguna and in the Tenerife Island Council, the INGENIA project stands out as an *avant-garde* initiative that merges research and innovation in a collective effort to address contemporary challenges in various scopes. In constant evolution since its inception, this multidisciplinary project represents a fundamental cornerstone in the university’s contribution to knowledge advancement and sustainable development. INGENIA’s heart beats after ambitious objectives, ranging from basic research drive to practical application of solutions in everyday life. Its mission core is to attain specific goals, such as discovering new scientific knowledge, creating innovating technologies or improving social and health services.

INGENIA’s strength lies in its interdisciplinary approach, encompassing varied fields such as Science, Technology, Engineering and Social Sciences. This knowledge mix allows researchers to face problems from different perspectives, fostering comprehensive understanding and generating holistic solutions. The methodology employed in INGENIA is characterised by its scientific rigour and for aiming at tangible results. From theoretical research to practical implementation, researchers use advanced tools and *avant-garde* technologies to materialise their ideas and address complex problems in an effective way. One of INGENIA’s fundamental cornerstones is its commitment to positive social impacts. The solutions and advances developed not only seek to contribute to enhancing knowledge but also to improve quality of life in local and global communities. Technology transfer and constant dialogue with society are key elements to ensure that the results benefit the highest number of people possible.

INGENIA is built on the premise that collaboration is the key to success. Therefore, it forges strategic alliances with other academic institutions, governmental bodies, the industry sector and not-for-profit organisations. These collaborative initiatives strengthen the project’s ability to address complex problems, leveraging diversity in terms of skills and perspectives. Throughout its existence, INGENIA has accrued an impressive set of achievements, which can manifest themselves in the form of high-impact academic publications, patents, innovating technological developments, contributions to public policies and perceptible changes in society or industry. The project is not merely about immediate results but also about the long-lasting legacy it leaves in the academic and social panorama. In short, the INGENIA project by the University of La Laguna is a collaboration and innovation beacon, representing the institution’s constant dedication to excellence in research and to exerting positive effects on society. Its multidisciplinary approach and prominent achievements turn it into an exemplary referent in the academic and scientific scope^(1,4)^.

## OBJECTIVE

To assess the educational actions proposed in the field of healthy lifestyles developed both by the network’s Primary Health Care professionals from the Canary Health Service and by the teaching staff of the educational centres involved, with the help of undergraduate Nursing students in the Service-Learning (S-L) modality.

## METHOD

Designed according to a pre-/post-test methodological scheme, this intervention project was implemented within the scope of ‘‘INGENIA. Agents of change for the SDGs’, 2^nd^ Edition. This initiative was generously sponsored by the University of La Laguna and by the Tenerife Island Council and was developed between February and June 2021.

The impact exerted by the intervention was assessed by applying a questionnaire at two key moments: at the beginning of the project (pre-test) and three months after it had ended (post-test). In both instances, the participants answered the Questionnaire on Health Habits related to Overweight/Obesity in childhood (*Cuestionario de Hábitos de Salud relacionados con el Sobrepeso/Obesidad infantil*, CHS-SO), an instrument designed and validated by Varela in 2013. This questionnaire allowed obtaining detailed quantitative data about the participants’ health habits.

The analysis regarding the impact exerted by the project was grounded on a direct comparison between the data collected in the initial phase and those obtained once the educational intervention had ended. This longitudinal comparison enabled determining the changes produced in the participants’ health habits as a result of the intervention.

In addition, in order to have a comprehensive view of the experience, a satisfaction survey was applied both to the students that actively took part in the intervention and to the teachers that collaborated in its development. The questions in this survey were formulated using a Likert-type scale. This scaled answer format allowed quantifying the participants’ and faculty’s perceptions and assessments in relation to various aspects of the project, providing valuable information about their satisfaction levels and the perceived effectiveness of the intervention.

## RESULTS

### Learning and overcomings

The proposal in this project was focused on comprehensively implementing an educational intervention in a number of educational centres with primary education pupils, in which the objective was that ‘those who are children now become healthy adults in the future’. Currently, some members of our society (the Spanish one) are unaware of the optimal habits to strike a balance between the different spheres that shape them as individuals, namely: psychological, social and spiritual.

This aims at benefiting the quality of life of these ‘future citizens’, thus adopting healthy habits that will assist in social integration, tolerance and all those ingredients that are necessary for self-fulfilment as a person within a given society.

Those in charge of developing the intervention were ‘volunteer Nursing students’ attending second year onwards from the undergraduate course, with all the precise knowledge required to implement the aforementioned educational intervention, namely: health education, health promotion and a helping relationship.

When it came to implementing the training actions, educational centres from the Tenerife island that wished to collaborate in the project were selected. The situation underwent at the global level (specifically in Spain) regarding the COVID-19 pandemic required setting a clearly defined strategy. A series of activities was proposed to run the programme, namely: selecting the educational centres; contacting all of them; recruiting the volunteer students and organising the educational intervention; buying and managing the resources; assessing the educational intervention at the end; and meeting the teaching professionals involved. A number of activities with the primary education pupils were added to the aforementioned, such as: assessing the educational intervention at the beginning; allocating the pupils and participants to individual and in-person meetings or, depending on the health situation, in the remote modality; meetings in small groups or through remote means (depending on possible social distancing measures); group meetings, in open spaces or telematically; and healthy breakfasts/tea snacks, either in groups or individually and in the open air.

We should take into account that the educational intervention was focused on the participants acquiring tools that would allow them to improve their health, enhancing their abilities and autonomy and fostering healthy relationships with healthy behaviours, always centred on sustainable development goals 3 (Health and well-being) and 4 (Quality education). A minimum of 2 scheduled contact instances between the Nursing students and the pupils attending the educational centres was established. These contacts lasted 2 hours each and were developed as follows: first contact with the participants (primary education pupils) in a neutral and social setting that was pleasant and comfortable. An initial assessment was made to know to what extent the educational intervention should be implemented (it is to be recalled that contact with the participants was proposed in a neutral setting following the distancing and safety measures due to the pandemic situation). Activities promoting the most suitable health-related changes for each group of primary education pupils were initiated: the importance of physical exercise; what I should eat to follow a balanced diet in line to my nutritional requirements; what should I play with friends and family members; the need to eliminate addictive behaviours such as alcohol or tobacco consumption (recommendations about healthy habits during confinement were also offered, providing suggestions regarding activities to perform in that case); a meeting was organised with the entire group where the pupils along with the Nursing students engaged in group games and dynamics that fostered an interaction targeted at improving healthy habits (the educational centre yard was arranged in a way so as to comply with the hygiene measures recommended by the health agents); a healthy tea snack was organised in the last meeting at an open-air park, along with leisure activities such as tabletop/card games or handicrafts, among others, aimed at strengthening the concepts acquired; finally, a meeting was held with the professionals involved and the educational centres’ faculty to inform them about the project results and the health assets map (it was necessary to hold this meeting online in several occasions due to worsening of the pandemic).

The programme was a success: it enhanced the primary education pupils’ knowledge about healthy habits and reduced health and social costs in the long run, thanks to the participation of undergraduate Nursing students from the Nuestra Señora de Candelaria Nursing School (*Escuela de Enfermería de Nuestra Señora de Candelaria*, EUENSC), attached to the University of La Laguna. The results obtained show high values both in satisfaction and in knowledge acquisition among the primary education pupils with whom the training intervention was performed.

## EXPERIENCES

The interaction between the undergraduate Nursing students from EUENSC and the primary education pupils gave rise to a considerable increase in the former’s motivation after implementing the training actions performed in the S-L modality. Likewise, the faculty acting as mentors in the project showed high satisfaction levels in developing this joint activity with the undergraduate students, creating a strong relationship that lasts to the present day (the students that took part in this project are already graduates).

### Study limitations

It is to be noted that the “Experience report” proposal type presents limitations as for its reproduction. This is solely the experience underwent by the author, who took part in a teaching innovation project of the S-L type where learnings, overcomings and experiences are shared based on the senses and meanings assigned by those involved. Therefore, this text should be interpreted as the description of an experience, namely: a human experience.

## FINAL CONSIDERATIONS

S-L is an educational methodology that combines learning and service processes for the community in an integrated manner. Instead of focusing exclusively on theoretical knowledge acquisition in a traditional academic environment, S-L seeks to link learning with the practical application of that knowledge in real-life situations, with the purpose of addressing actual community needs. This type of teaching methodology can be implemented in various contexts, such as schools, universities and other educational settings. It can address a broad range of topics, from education and health to environment and social justice. This methodology seeks to train committed citizens that are fully aware of their ability to contribute to society well-being.

It is evident that there is an important increase in satisfaction, both among the students and among the faculty that employs this teaching methodology, in addition to the benefits it offers to the society they live in. It is worth noting that the educational centres requested us to devise training actions targeted at improving sleep quality and at optimal handling of mobile devices among the primary education pupils. The ‘Healthy Future’ project has met the expectations that were set out when designing it, thus opening new paths for future proposals.

## Data Availability

It can be consulted on the website of the University of La Laguna, INGENIA subpage (https://www.ull.es/portal/ingenia/proyectos-ingenia-vi/#).
