# Supplementary material for: The INGENIA project and “Healthy Future”: a multidisciplinary innovative impulse within the SDGs
Source: Rev Bras Enferm. 2025 Dec 8;78(Suppl 3):e20240039. doi: 10.1590/0034-7167-2024-0039 (PMC12707770; doi:10.1590/0034-7167-2024-0039)
Supplement: Translation

# Translation: El proyecto INGENIA y ‘Futuro saludable’: un impulso innovador multidisciplinar dentro de los ODS

**DOI:** 10.1590/0034-7167-2024-0039es

**Authors:** Francisco Javier Castro-Molina, Gilberto Tadeu Reis da Silva, Sagrario Gómez-Cantarino, Jesús Manuel García-Acosta

**Affiliations:** I Universidad de La Laguna, Escuela Enfermería Nuestra Señora de Candelaria. Islas Canarias, España; II Universidade Federal da Bahia, Salvador, Bahía, Brasil; III Universidad de Castilla La Mancha. Toledo, España

**Keywords:** Aprendizaje, Estilo de Vida Saludable, Estudiantes, Docentes, Educación Primaria y Secundaria.

## Abstract

**Objetivo:**

evaluar las acciones educativas propuestas en el campo del estilo de vida saludable desarrollada desde el Servicio Canario de Salud y por el profesorado de los centros educativos, de la mano de estudiantes del grado de Enfermería empleando Aprendizaje-Servicio.

**Método:**

proyecto de intervención con un pretest-postest que se desarrolló de la II Edición de ‘INGENIA. Agentes del cambio por los ODS’, entre febrero y julio de 2021. Se procede a medir impacto tanto de la intervención como de la satisfacción de los implicados.

**Resultados:**

El programa fue un éxito, aumentando tanto los conocimientos en hábitos saludables de los alumnos de educación primaria como la satisfacción de los agentes ejecutores del programa.

**Conclusiones finales:**

destaca el aumento considerable de la satisfacción tanto del alumnado como del profesorado que emplea esta metodología docente, además de los beneficios que genera a la sociedad en la que están insertos.

## INTRODUCCIÓN

La Organización Mundial de la Salud (OMS) define la salud como “el completo estado de bienestar físico, psíquico y social, y no solo la ausencia de enfermedad o invalidez”. Así, hoy por hoy hablamos de estilo de vida saludable, dejando atrás el trasnochado concepto de vida sana, atendiendo a aspectos fundamentales tales como el ejercicio físico, la alimentación, las relaciones con el entorno más próximo y las relaciones que mantenemos dentro del nicho social que ocupamos. Pero ¿qué definimos como estilo de vida saludable? En tres palabras: vivir en armonía. Es decir, disfrutar la mejor calidad de vida que esté a nuestro alcance. Situaciones como la ingesta equilibrada, el ejercicio habitual, presentar un índice de masa corporal (IMC) saludable, evitar las sustancias tóxicas, el ‘equilibrio mental’ y dormir las horas necesarias se convierten en los ingredientes necesarios para poder alcanzarla^(1-3)^.

La salud, tanto de niños como de niñas, es fundamental para que en un futuro puedan tener una vida plena que permita que logren su autorrealización. La salud infantil se sustenta en tres elementos básicos: la detección temprana de problemas en su desarrollo, la prevención de cualquiera de las enfermedades contagiosas que puedan surgir y la promoción de hábitos saludables mediante intervenciones donde se fomente la educación para la salud (EpS). Así, la infancia es un momento primordial para el aprendizaje donde el niño y la niña pueden adquirir fácilmente hábitos saludables que con seguridad se conservan hasta la adultez. Además, debe resaltarse que los hábitos saludables previenen enfermedades que surgirán con el paso de los años, ayudando a dar equilibrio desde esferas como la física, la psíquica, la social o la espiritual. En la isla de Tenerife existen numerosas iniciativas desde la red de Atención Primaria que buscan cubrir las necesidades de la población. En el caso de los niños, en ocasiones, requieren de intervenciones educativas cercanas, dentro del espacio académicos de los centros educativos, en el que se les debe instruir en los hábitos básicos que faciliten la instalación de un estilo de vida saludable^(2-6)^.

El proyecto INGENIA, desarrollado en la Universidad de La Laguna y el Cabildo Insular de Tenerife, destaca como una iniciativa vanguardista que une la investigación y la innovación en un esfuerzo colectivo para abordar desafíos contemporáneos en diversos ámbitos. Este proyecto multidisciplinario, que ha venido evolucionando desde su inicio, representa un pilar fundamental en la contribución de la universidad al avance del conocimiento y al desarrollo sostenible. El corazón de INGENIA late con objetivos ambiciosos, que van desde el impulso de la investigación básica hasta la aplicación práctica de soluciones en la vida cotidiana. Alcanzar metas específicas, como el descubrimiento de nuevos conocimientos científicos, la creación de tecnologías innovadoras o la mejora de servicios sociales y sanitarios, constituye el núcleo de su misión.

La fortaleza de INGENIA radica en su enfoque interdisciplinario, abarcando campos tan diversos como la ciencia, la tecnología, la ingeniería y las ciencias sociales. Esta amalgama de conocimientos permite a los investigadores enfrentar problemas desde diversas perspectivas, fomentando una comprensión integral y la generación de soluciones holísticas. La metodología empleada en INGENIA se caracteriza por su rigor científico y su orientación a resultados tangibles. Desde la investigación teórica hasta la implementación práctica, los investigadores utilizan herramientas avanzadas y tecnologías de vanguardia para materializar sus ideas y abordar problemas complejos de manera efectiva. Uno de los pilares fundamentales de INGENIA es su compromiso con el impacto social positivo. Las soluciones y avances desarrollados buscan no solo contribuir al avance del conocimiento, sino también mejorar la calidad de vida de las comunidades locales y globales. La transferencia de tecnología y el diálogo constante con la sociedad son elementos clave para garantizar que los resultados beneficien a la mayor cantidad de personas posible.

INGENIA se erige sobre la premisa de que la colaboración es la clave para el éxito. Por lo tanto, establece alianzas estratégicas con otras instituciones académicas, entidades gubernamentales, la industria y organizaciones sin fines de lucro. Estas colaboraciones fortalecen la capacidad del proyecto para abordar problemas complejos, aprovechando la diversidad de habilidades y perspectivas. A lo largo de su existencia, INGENIA ha acumulado un impresionante conjunto de logros, que pueden manifestarse en publicaciones académicas de alto impacto, patentes, desarrollos tecnológicos innovadores, contribuciones a políticas públicas y cambios perceptibles en la sociedad o la industria. El proyecto no solo se trata de resultados inmediatos, sino también del legado duradero que deja en el panorama académico y social. En resumen, el proyecto INGENIA de la Universidad de La Laguna es un faro de innovación y colaboración, representando la dedicación constante de la institución a la excelencia en la investigación y al impacto positivo en la sociedad. Su enfoque multidisciplinario y sus logros destacados lo convierten en un referente ejemplar en el panorama académico y científico^(1,4)^.

## OBJETIVO

Evaluar las acciones educativas propuestas en el campo del estilo de vida saludable desarrollada tanto por los profesionales de Atención Primaria de la red del Servicio Canario de Salud como por el profesorado de los centros educativos, de la mano de estudiantes del grado de Enfermería mediante la modalidad de Aprendizaje-Servicio (ApS).

## MÉTODO

Este proyecto de intervención, diseñado bajo un esquema metodológico pretest-postest, se implementó en el marco de la II Edición de ‘INGENIA. Agentes del cambio por los ODS’. Esta iniciativa contó con el generoso patrocinio de la Universidad de La Laguna y el Cabildo Insular de Tenerife, desarrollándose durante el periodo comprendido entre febrero y julio de 2021.

La evaluación del impacto de la intervención se llevó a cabo a través de la aplicación de cuestionarios en dos momentos clave: al inicio del proyecto (pretest) y tres meses después de su finalización (postest). En ambas instancias, los participantes completaron el cuestionario de *Hábitos de Salud relacionados con el Sobrepeso/Obesidad infantil (CHS-SO)*, un instrumento validado y diseñado por Varela en 2013. Este cuestionario permitió obtener datos cuantitativos detallados sobre los hábitos de salud de los participantes.

El análisis del impacto del proyecto se fundamentó en la comparación directa de los datos recogidos en la fase inicial con los datos obtenidos tras la conclusión de la intervención educativa. Esta comparación longitudinal posibilitó la determinación de los cambios producidos en los hábitos de salud de los participantes como resultado de la intervención.

Adicionalmente, con el objetivo de obtener una visión integral de la experiencia, se administró una encuesta de satisfacción tanto a los estudiantes que participaron activamente en la intervención como a los profesores que colaboraron en su desarrollo. Las preguntas de esta encuesta fueron formuladas utilizando una escala tipo Likert. Este formato de respuesta graduada permitió cuantificar las percepciones y valoraciones de los participantes y el profesorado en relación con diversos aspectos del proyecto, proporcionando información valiosa sobre su nivel de satisfacción y la efectividad percibida de la intervención.

## RESULTADOS

### Aprendizaje y superaciones

La propuesta de este proyecto se centró en la realización de una intervención educativa en un centro educativo con alumnos de educación primaria, de manera integral, en la que se tuvo como objetivo que ‘los que ahora son niños sean adultos saludables’. En la actualidad, algunos miembros de nuestra sociedad, la española, desconocen cuáles son los hábitos óptimos para lograr un equilibrio entre las diferentes esferas que lo conforman como persona: la esfera física, la esfera psicológica, la esfera social y la esfera espiritual.

Con ello se busca que se favorezca la calidad de vida de estos ‘futuros ciudadanos’, adquiriendo así hábitos saludables que ayudarán a la integración social, la tolerancia y todos aquellos ingredientes que se precisan para la autorrealización como persona perteneciente a una sociedad.

Los responsables de desarrollar la intervención fueron ‘estudiantes de enfermería voluntarios’, a partir del segundo año del grado de Enfermería, que dispusieron de todos aquellos conocimientos precisos para realizar la mencionada intervención educativa como la educación para la salud, la promoción de la salud y la relación de ayuda.

A la hora de impartir las acciones formativas, se seleccionaron centros educativos de la isla de Tenerife que quisieron colaborar con el proyecto. La situación que se vivió a nivel mundial, y en concreto en España, con respecto a la pandemia sufrida por el COVID 19, obligó a marcar una estrategia claramente definida. Para llevar a cabo el programa, se plantearon una serie de actividades como: seleccionar los centros educativos; el contactar con todos ellos; la captación del alumnado voluntario y la organización de la intervención educativa; la adquisición y la gestión de los recursos; la realización de la valoración de la intervención educativa finales; y la reunión con los profesionales docentes implicados. A ello se le unieron actividades con los alumnos de educación primaria tales como: la realización de la valoración de la intervención educativa inicial; la asignación de alumnos y de los participantes en reuniones individuales y personales o, dependiendo de la situación sanitaria, telemática; reuniones en grupo pequeño o por medios telemáticos (dependiendo de posibles distanciamiento social); reuniones grupales, en espacios abiertos o telemáticamente; desayuno/merienda saludable, grupal o individual, en espacios aireados.

Debemos tener presente que la intervención educativa se centró en que los participantes adquieran herramientas que le permitieran mejorar su salud, potenciando sus capacidades y autonomía, fomentando sus relaciones sanas en las que estuvieran presentes conductas saludables, centrados siempre en los objetivos de desarrollo sostenible 3 (salud y bienestar) y 4 (educación de calidad). Se estableció un mínimo de 2 contactos programados entre el estudiante de enfermería y los alumnos del centro educativo, de aproximadamente 2 horas cada uno, que se desarrolló de la siguiente manera: primer contacto con los participantes (alumnos de educación primaria) en un entorno neutro y social que fue cómodo y confortable. Se realizó una valoración inicial para conocer hasta que medida se debía realizar la intervención educativa (recordar que por la situación de pandemia se planteó un contacto con los participantes en entorno neutro siguiendo las recomendaciones de distanciamiento y de seguridad); se iniciaron actividades que promovieran los cambios de salud más adecuados para cada grupo de alumnos de educación primaria: la importancia del ejercicio físico, qué debemos comer para tener una dieta equilibrada adecuadas a sus necesidades nutricionales, a qué debo jugar con mis amigos y familiares, la necesidad de eliminar conductas adictivas como el alcohol o el tabaco, por ejemplo (igualmente se realizaron recomendaciones sobre hábitos saludables en confinamiento, dando sugerencias en torno a las actividades a realizar en caso de que este suceda); se organizó una reunión de todo el grupo en la que los alumnos acompañados de los estudiantes de enfermería realizarán juegos y dinámicas grupales que fomentaran una interacción orientada a mejorar los hábitos saludables (se dispuso el patio del centro educativo para mantener las medidas de higiene recomendadas por los agentes sanitarios); en la última sesión se organizó una merienda saludable en un parque al aire libre, junto a actividades de ocio como juegos de mesas, cartas o manualidades entre otros, que estuvieron orientados a fortalecer los conceptos adquiridos; finalmente se procedió a realizar una reunión con los profesionales implicados y profesores de los centros educativos para informar sobre los resultados del proyecto y del mapa de activos de salud (en varias ocasiones hubo que realizarse online ante la situación de agravamiento de la pandemia).

El programa fue un éxito, aumentando los conocimientos en hábitos saludables de los alumnos de educación primaria (6 a 12 años), reduciendo costes sanitarios y sociales a la larga, gracias a la intervención de alumnos del grado de Enfermería de la Escuela de Enfermería de Nuestra Señora de Candelaria (EUENSC) adscrita a la Universidad de La Laguna. Los resultados obtenidos muestran valores altos tanto de satisfacción como de adquisición de conocimientos del alumnado de educación primaria al que se le realizó la intervención formativa.

### Vivencias

La interacción del alumnado del grado de Enfermería de la EUENSC con el alumnado de educación primaria generó un considerable aumento de la motivación del primero tras la realización de las acciones formativas realizadas dentro de la modalidad de ApS. Igualmente, el profesorado que actuó de mentor dentro del proyecto manifestó altos índices de satisfacción en el desarrollo de esta actividad conjunta con el alumnado de grado, creando una relación fuerte que en la actualidad perdura (actualmente el alumnado que participó en este proyecto ya está graduado).

### Limitaciones del estudio

Es de destacar que el tipo de propuesta “informe de experiencia” presenta limitaciones en cuanto a su reproducción. Se trata estrictamente de la experiencia del autor involucrado en un proyecto de innovación docente tipo ApS, donde se comparten aprendizajes, superaciones y experiencias, desde el sentidos y significados atribuidos por quienes lo experimentan. Así, se debe contemplar este discurso como una descripción de una experiencia, es decir, la experiencia humana

## CONSIDERACIONES FINALES

El ApS es una metodología educativa que combina procesos de aprendizaje y de servicio a la comunidad de manera integrada. En lugar de centrarse exclusivamente en la adquisición de conocimientos teóricos en un entorno académico tradicional, el ApS busca vincular el aprendizaje con la aplicación práctica de esos conocimientos en situaciones del mundo real, con el propósito de abordar necesidades reales de la comunidad. Este tipo de metodología docente puede llevarse a cabo en diversos contextos, incluyendo escuelas, universidades, y otros entornos educativos. Puede abordar una amplia gama de temas, desde educación y salud hasta medio ambiente y justicia social. Esta metodología busca formar ciudadanos comprometidos y conscientes de su capacidad para contribuir al bienestar de la sociedad.

Queda claro que existe un aumento importante de la satisfacción tanto del alumnado como del profesorado que emplea esta metodología docente, además de los beneficios que genera a la sociedad en la que están insertos. Cabe destacar que desde los centros educativos se nos solicitó la creación de acciones formativas que fueran orientadas hacia la mejora de la calidad de sueño y un manejo optimo de los dispositivos móviles por parte del alumnado de educación primaria. El proyecto ‘Futuro saludable’ ha cubierto las expectativas que se plantearon a la hora de su diseño, abriendo nuevos caminos para futuras propuestas.

## Data Availability

Puede ser consultado en la web de la Universidad de La Laguna, subpágina web INGENIA (https://www.ull.es/portal/ingenia/proyectos-ingenia-vi/#).
